# Neosaxitoxin Downregulates Inflammation in an Equine In Vivo Model of Osteoarthritis

**DOI:** 10.3390/biom16081142

**Published:** 2026-08-06

**Authors:** Cristóbal Dörner, Néstor Lagos, Lissette Oyaneder, Carlos González, Galia Ramírez-Toloza, Bruno C. Menarim

**Affiliations:** 1 Escuela de Medicina Veterinaria, Sede Viña del Mar, Facultad de Ciencias de la Vida, Universidad Andres Bello, Quillota 980, Viña del Mar 2520000, Chile; 2 Programa de Doctorado en Ciencias Silvoagropecuarias y Veterinarias, Campus Sur, Universidad de Chile, Santa Rosa 11315, Santiago 8820808, Chile; 3 Membrane Biochemistry Laboratory, Department of Physiology and Biophysics, Faculty of Medicine, University of Chile, Independencia 1027, Santiago 8380000, Chile; nlagosw@gmail.com; 4 Equestria Equine Medical Center, Quillota 2260000, Chile; lissette.oyaneder@ug.uchile.cl; 5 Escuela de Medicina Veterinaria, Sede Santiago, Facultad de Ciencias de la Vida, Universidad Andres Bello, República 440, Santiago 8320000, Chile; carlosgonzalez@unab.cl; 6 Department of Animal Preventive Medicine, Faculty of Veterinary Medicine, University of Chile, Santa Rosa 11735, Santiago 6640022, Chile; galiaram@uchile.cl; 7 Gluck Equine Research Center, Department of Veterinary Sciences, Martin-Gatton College of Agriculture, Food and Environment, University of Kentucky, Lexington, KY 40546, USA

**Keywords:** horse, arthropathies, arthritis treatment, neosaxitoxin, homeostasis

## Abstract

Chronic synovial inflammation is a hallmark of osteoarthritis progression and is tightly regulated by synovial macrophages. Recently, voltage-gated sodium channels (NaV) have emerged as potent modulators of macrophage-driven inflammation, positioning them as novel therapeutic targets. Among selective NaV channel blockers, neosaxitoxin exerts remarkable anesthetic and immunomodulatory effects; however, its effects on joint inflammation upon intra-articular delivery remain unexplored. Using an equine model of bilateral carpal osteoarthritis, this study evaluated the immunomodulatory and tissue-preserving effects of intra-articular neosaxitoxin. Sixteen horses were randomized into two experimental groups (n = 8/each): Neosaxitoxin in one joint and triamcinolone (+control) in the contralateral joint; or neosaxitoxin in one joint and saline (−control) in the contralateral joint. Clinical parameters, synovial fluid cytology and cytokine profiles, and histological changes in synovium and cartilage were assessed over 30 days. Neosaxitoxin reduced synovial inflammation, evidenced by decreased synovial effusion and surface temperature, along with improved joint flexion. Furthermore, synovial fluid from neosaxitoxin-treated joints exhibited lower counts of erythrocytes, neutrophils, total protein, and key pro-inflammatory mediators (IL-1β and IL-6) compared to saline-treated controls. Histologically, neosaxitoxin-treated joints exhibited modest synovial inflammatory cell infiltration and minor cartilage abnormalities. In contrast, control joints exhibited synovial hyperplasia, fibrovascular proliferation, and cartilage degeneration. Our data suggests that intra-articular neosaxitoxin better preserved joint homeostasis by limiting synovial inflammation and cartilage damage. These results warrant further investigation on Neosaxitoxin as a candidate treatment for inflammatory arthropathies.

## 1. Introduction

Osteoarthritis (OA) is a debilitating disease that affects over 30% of the global population [1]. OA remains a major public health concern and represents the second most costly condition in the United States healthcare system [1,2]. OA is also a highly prevalent condition in domestic animals, and the leading cause of lameness in equine athletes, translating into a substantial economic burden to the equine industry [3,4,5,6]. Joint inflammation is a central feature of OA progression and is primarily regulated by synovial macrophages [7,8,9,10,11,12,13]. Upon tissue injuries that exceed their homeostatic functions, synovial macrophages incite an inflammatory response to counteract damage and ultimately resolve inflammation [11,12,13,14]. However, due to a multitude of unknown reasons, the resolution phase of this process often fails, resulting in chronic inflammation as seen in OA [15,16,17,18]. Current OA therapies provide short-term relief but fail to resolve joint inflammation long- term and often have negative side effects [19]. There is a critical need for innovative therapies that can provide lasting clinical improvements while restoring joint homeostasis.

Voltage-gated sodium channels (NaV) are well known for controlling action potential in excitable cells such as neurons and myocytes [20]. In addition, there is growing evidence of their role in the activation of immune cells during inflammatory responses including macrophages [20,21,22,23,24,25,26]. There is growing evidence the by controlling intracellular Na^+^ and Ca^2+^ homeostasis, NaV channels also regulate NF-κB activation and the expression of inflammatory mediators providing a biological rationale for investigating their role in the regulation of synovial inflammation [27,28,29,30,31,32,33].

Neosaxitoxin (NeoSTX) is a phycotoxin with high capacity to block NaV channels and has mainly been studied for its local anesthetic properties [34,35,36,37]. Our previous in vitro study on NaV channels in the inflammatory response of macrophages [33] suggested that targeted blocking of these channels with NeoSTX may reduce joint inflammation and potentially enhance their pro-resolving functions [37,38]. Altogether, these observations pose NaV channels as candidate new therapeutics in OA. The aim of this study was to investigate the immune-modulatory effects of joint injection with NeoSTX, in an equine experimental model of OA. We hypothesized that Neo-STX would modulate inflammation, enhancing mechanisms of synovial homeostasis and mitigating OA progression.

## 2. Materials and Methods

### 2.1. Study Design

Sixteen crossbred horses (9 geldings and 7 mares) with a median age of 10.5 years of age (range, 6–14) and a median body weight of 375 kg (range, 315–440) were subjected to a carpal osteochondral fragment model of OA [39]. The protocol was prepared before this study, registered, and approved by the “Comité Institucional de Cuidado y Uso de Animales” of the University of Chile (CICUA, certificate no.: 23716–VET–UCH1e, approved date: 8 October 2024)). The study was conducted at the College of Veterinary and Animal Sciences–University of Chile and the Equestria Equine Medical Center, Universidad Andrés Bello.

**Figure 1:**
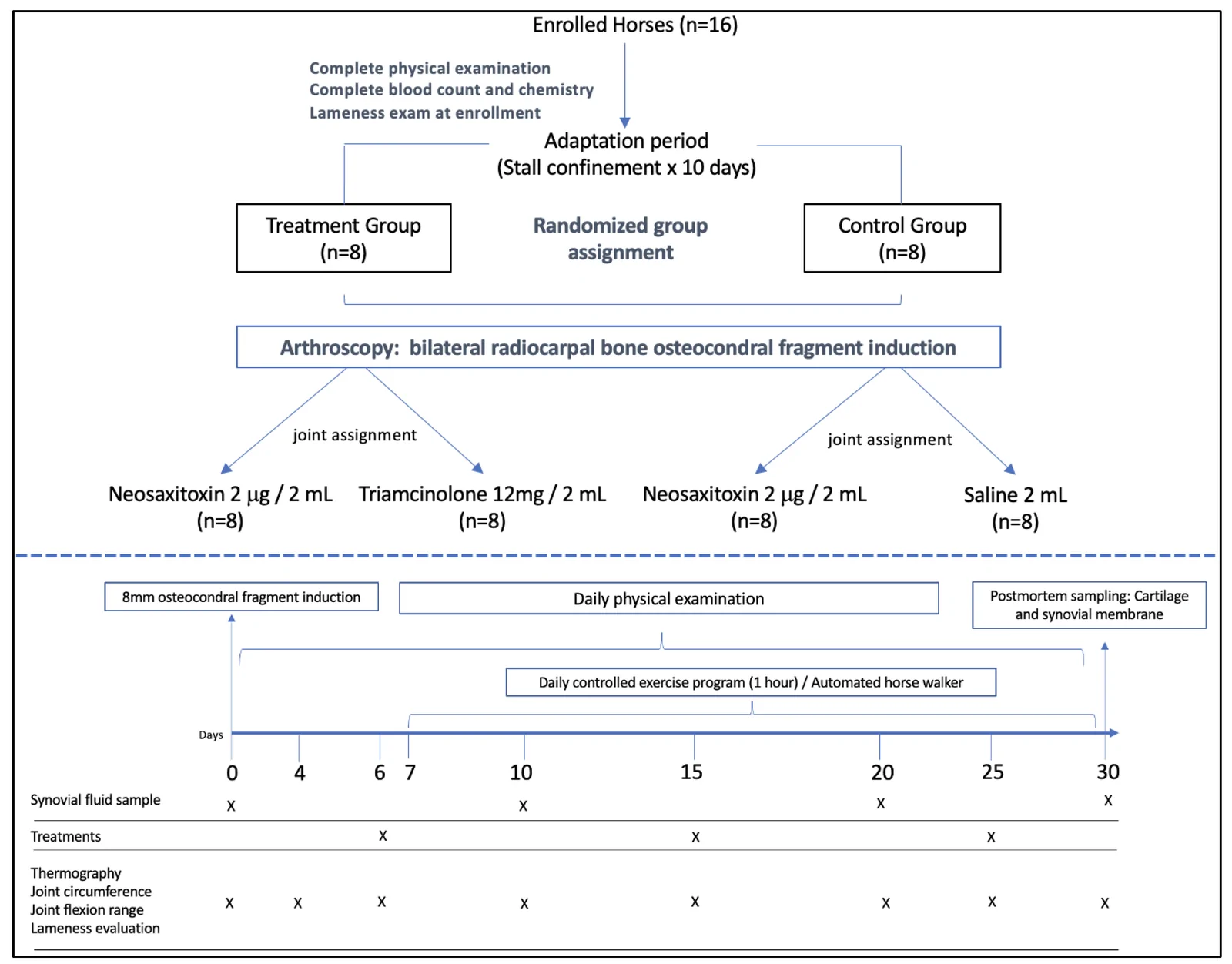
Schematic outlining the study design, treatment assignment and timing for model induction and sampling.

To ensure general and musculoskeletal health at enrollment, horses included in this study had normal cell blood counts (CBC) and blood chemistry. All horses underwent a complete musculoskeletal exam, including response to joint flexion, gait analysis and diagnostic imaging, and were identified to be free of lameness and musculoskeletal pathology. A simple sequential randomization method was employed for treatment assignment prior to enrollment of the horses, ensuring an equal number of animals per treatment group. The timeline for the study including horses’ enrollment, model induction, treatment and sample collection is detailed in Figure 1. All horses underwent bilateral middle carpal arthroscopy to further confirm joint health and to create the carpal osteochondral fragment model of OA as previously described [39,40]. Horses were allocated in two groups (n = 8 horses/each). The treatment group consisted of five geldings and three mares. For each horse, one joint was randomly assigned to receive an intra-articular injection of NeoSTX (Figure 1), while the contralateral joint received triamcinolone (TA) as a positive control [41,42]. The control group comprised four geldings and four mares, which were treated with NeoSTX in one joint and saline as a negative (vehicle) control in the contralateral joint. Only the first author (CD) was aware of the assigned treatments. Clinical assessments recorded by a separate, blinded evaluator (LO).

The outcome measures used to assess treatment effect included synovial fluid cytology, cytokine/chemokine profiling, and histological evaluation of the synovial membrane and articular cartilage. Additional outcome evaluated included thermographic assessment of joint surface, maximal joint flexion angle, and joint circumference (Figure 1).

### 2.2. Animal Husbandry

Horses were housed in 10′ × 10′ stalls with shavings and fed free choice alfalfa-based hay and fresh water. Horses were stall-confined for a total period of 40 days, including: 10 days of adaptation, followed by 30 days of exercise program. Throughout the study period, 7 days after surgery horses were turned out for 4 h daily in small paddocks (20′ × 20′).

### 2.3. OA Model

On day 0 horses underwent bilateral arthroscopy of the middle carpal joints under general anesthesia. Both middle carpal joints were thoroughly examined to rule out pre-existing abnormalities. An osteochondral fragment was created in both middle-carpal joints at the radial carpal bone (RCB), at the level of the medial synovial plica [39]. The fragment was allowed to remain adhered to the joint capsule proximally, and the exposed subchondral bone between the fragment and parent bone was debrided using a motorized arthroburr, creating a 15 mm wide defect in which the bone fragment sat. The skin was closed with 2–0 glyconate monofilament in a simple interrupted pattern. Both operated joints received compressive bandages. Since joint inflammation modulation was a primary outcome of the study, horses did not receive NSAIDs nor antimicrobials following the aseptic surgical procedure. Bandages were changed every 3 days or when data collection was required but maintained until suture removal at 14 days after surgery. After 7 days of creation of the osteochondral fragments, horses entered a daily exercise regimen of 7 days/week for 3 more weeks. Each day, horses were trotted for 1 h in an automated equine exerciser at 3.2 m/s (14 m diameter) [43]. This modification from the original protocol [43] was designed to provide consistent and repetitive joint loading sufficient to induce synovial inflammation while ensuring animal safety.

### 2.4. General and Musculoskeletal Evaluation

**Figure 2:**
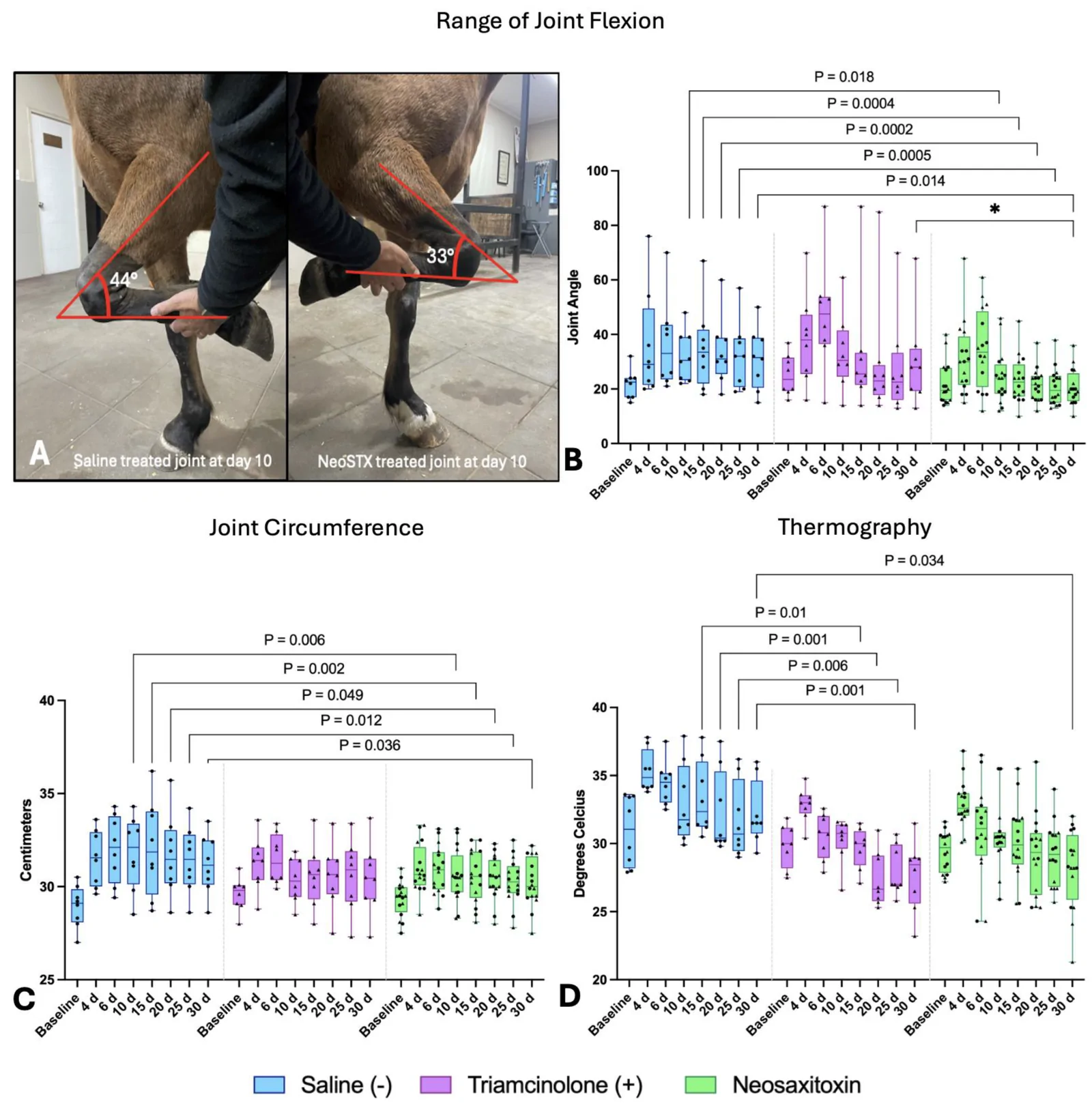
Clinical parameters following intra-articular treatments. Representative images of differences in joint flexion range between joints treated with saline and NeoSTX at day 10, respectively (**A**). Boxplots of the results with boxes representing interquartile range with a median and whiskers representing range, show changes in joint flexion angle (**B**), joint circumference (**C**), and joint surface temperature (**D**), at baseline and during the 30-day follow-up. NeoSTX and TA improved joint mobility, reduced swelling, and decreased surface temperature, whereas saline-treated horses showed persistently reduced flexion, a greater joint circumference, and elevated temperature. * Statistical trend between NeoSTX and TA (*p* = 0.1). *p*-value = 0.05. (▲) represents individual data points from the NeoSTX vs. TA comparison, and (●) represents individual data points from the NeoSTX vs. saline comparison.

Clinical monitoring included daily assessment of heart rate (HR), respiratory rate (RR), rectal temperature, and feed and water intake. Joints were assessed for surface temperature using infrared thermography (Flir One^®^ camera), at days 0, 2, 4, 7, 10, 15, 20, 25, and 30. All thermographic measurements were conducted at room temperature in the same temperature-controlled room preserving a one-meter distance between the camera and the joint. All horses were acclimatized for 20 min prior to imaging. At the same time points described, inflammation was evaluated by measuring joint circumference, range of motion and pain response. Joint circumference was measured at a standardized anatomical site defined by clipped hair landmarks at the level of the middle-carpal joint, with the tape centered at the distal border of the accessory carpal bone. To assess pain response, the limb was manually supported and gently flexed until the horse exhibited a pain response of limb withdrawal. After identifying the point of maximal flexion, the flexion angle was measured using a digital goniometer with the longitudinal axes of the third metacarpal bone and the radius serving as anatomical reference (Figure 2A).

### 2.5. Joint Treatments, Synovial Fluid Sampling and Assessment

Neosaxitoxin was extracted from *Cyanobacteria aphanizomenon* sp. cultures and purified by high-performance liquid chromatography (HPLC) as previously described [44]. The toxin concentration was standardized at doses of 10 µg/mL in 0.9% saline solution. The selected dose of 20 µg of NeoSTX was calculated based on previous studies [37,45].

On days 6, 15 and 25 the assigned treatments were delivered using aseptic procedure (Figure 1). Treatments were randomly assigned to one experimental joint and maintained throughout the study. In the treatment group (NeoSTX vs. TA) one joint was randomly selected to be injected with 2 mL of NeoSTX (10 µg/mL 0.9% NaCl) and the contralateral joint with 2 mL of triamcinolone acetonide (6 mg/mL) [41,42]. For horses allocated in the control group, one joint was randomly selected to receive 2 mL of NeoSTX while the contralateral joint received 2 mL of saline as negative (vehicle) control. The treatment protocol (3 injections) was aimed at mimicking the treatment regimens commonly applied to the use of orthobiological therapies in joint disease [46,47].

Synovial fluid samples (2 mL) were aseptically collected from each treated joint at day 0 (baseline) 10, 20 and 30, and split into two aliquots: one without anticoagulant (Eppendorf^®^ Protein LoBind, Merck, Darmstadt, Germany) and another containing EDTA (Vacutainer™, Becton Dickinson, Franklin Lakes, NJ, USA). SF was first assessed for color, turbidity, and viscosity. EDTA-free samples were centrifuged at 2000× *g* for 10 min to remove cellular components. A 200 μL aliquot was immediately analyzed using a spectrophotometer (Chemo20^®^, Shinova, Shanghai, China) to measure total protein [48]. The remaining cell-free supernatants were stored at −20 °C for batch cytokine analyses. EDTA-treated samples were processed within 1 h using an automated hematology analyzer (Hemo Plus 2900V^®^, Shinova, Shanghai, China) to determine total nucleated cell counts and differential cell counts (neutrophils, mononuclear, and red blood cells).

### 2.6. Cytokine/Chemokine Quantifications

Synovial fluid concentrations of twenty-three analytes including cytokines, chemokines and growth factors were assessed using Luminex^®^ xMAP^®^ technology (Thermo Fischer Scientific, Waltham, MA, USA). All cytokine assays were performed by a commercial third-party laboratory in a blinded manner (Eve Technologies Corporation, Calgary, AB, Canada). Synovial fluid samples were hyaluronidase-digested (100 IU/mL testicular hyaluronidase in 0.05 M acetate buffer pH 4.5, LS005474; Worthington Biochemical, Lakewood, NJ, USA) using 10 µL of hyaluronidase solution per 200 µL of synovial fluid and incubated for 30 min at 37 °C [9]. Next, the multiplexing analysis was performed by Eve Technologies Corporation (Calgary, AB, Canada) using the Luminex^®^ 200™ system (Luminex Corporation/DiaSorin, Saluggia, Italy) with Bio-Plex Manager™ software version 6.2 (Bio-Rad Laboratories Inc., Hercules, CA, USA). The 23-Plex Discovery Assay ^®^ (MILLIPLEX^®^ Equine Cytokine/Chemokine Magnetic Bead Panel, EQCYTMAG-93K, MilliporeSigma, Burlington, MA, USA) was processed as per the manufacturer’s instructions and included the following targets: Eotaxin, FGF-2, Fractalkine, G-CSF, GM-CSF, GRO/KC, IFNγ, IL-1α, IL-1β, IL-2, IL-4, IL-5, IL-6, IL-8, IL-10, IL-12p70, IL-13, IL-17A, IL-18, IP-10, MCP-1, RANTES, and TNF-α.

### 2.7. Synovial Membrane and Cartilage Histology

At day 30, horses were humanely processed at a slaughterhouse, joints were evaluated and synovial membrane and osteochondral biopsies were harvested using a 6 mm dermal biopsy- and osteochondral punches. For histological assessment, tissue samples were fixed in acetic zinc formalin (AZF Fixative^®^, Newcomer Supply, Waunakee, WI, USA). Following fixation, osteochondral samples were decalcified in neutral 10% EDTA solution prior to processing. Tissues were paraffin embedded, sectioned at 5 µm and stained with hematoxylin and eosin (H&E). In addition to H&E, osteochondral samples were also stained with Safranin-O to assess proteoglycan content. Both synovium and osteochondral samples were blindly analyzed by a board-certified veterinary pathologist using the OARSI scoring guideline [49]. Samples were analyzed by the average of scores from five randomly selected fields per section at 200× magnification. Histopathological data were available for 10 of the 16 horses included in the study. Tissue collection could not be completed for the remaining horses due to logistical constraints at the slaughterhouse. No imputation of missing data was performed. Analyses were therefore conducted using complete-case analysis on the available samples (n = 10 horses; 5/group).

### 2.8. Statistical Analysis

The sample size for this study (n = 16; 8 animals per group) was calculated using G*Power software version 3.1 (Düsseldorf, Germany) through an a priori power analysis, considering α, type II error, and effect size (f). Parameters included a 95% confidence level, a significance threshold of 0.05, and 80% statistical power (1–β) with an effect size f of 0.3. For quantitative variables, normality was assessed using the Shapiro–Wilk test. To further account for data’s structure, a multivariate approach was applied to identify relevant patterns. A Generalized Linear Mixed Model (GLMM) was fitted, with treatment, time, and their interaction as fixed effects, while horses were included as a random factor. Estimated marginal means (EMMs) were subsequently computed to facilitate pairwise comparisons between treatment groups (NeoSTX vs. TA and NeoSTX vs. saline) at each time point, as well as comparisons within each treatment group over time. Pairwise comparisons were adjusted using Tukey’s correction, and results are presented with 95% confidence intervals. For cytokine values that were below the lower limit of detection (LLOD), LLOD*0.5 imputed values were used in statistical analysis. Data obtained from histopathological samples were analyzed using the non-parametric Kruskal–Wallis test followed by a Pairwise Wilcoxon post hoc test using the Benjamini–Hochberg correction method. All statistical analyses were performed in R software version 4.0.2. Qualitative parameters were evaluated using the Pearson chi-square test. Statistical significance was set at *p* < 0.05.

## 3. Results

### 3.1. Clinical Evaluation: Joint Flexion Angle, Thermography and Circumference

All horses recovered from surgery uneventfully. On day 30, radiographs revealed varying levels of healing and subchondral sclerosis in the dorso-medial aspect of the radio-carpal bone (Figure S1). Induction of the model produced an inflammatory response characterized by increased joint flexion angle (decreased range of motion), joint circumference and temperature. Treatment with NeoSTX was associated with progressive improvement in joint flexion after day 10 when compared to saline (Figure 2A). Although no differences were detected between NeoSTX and TA, there was ultimately a trend of lower angles on NeoSTX-treated joints (* *p* = 0.1) (Figure 2B).

A similar pattern was observed for joint circumference, where from day 10 onwards NeoSTX-treated joints exhibited a sustained decrease in circumference, significantly lower than saline-treated joints, which remained increased following model induction. Contrarily, the circumference of NeoSTX- and TA-treated joints were largely comparable (Figure 2C).

The thermographic findings exhibited a similar pattern. Following an initial temperature increase associated with model induction, both NeoSTX and TA treatments led to a progressive decrease in joint temperature, yet significant differences for NeoSTX were only observed at day 30 (Figure 2D). In contrast, saline-treated joints maintained elevated temperatures throughout the study.

Collectively, these findings suggest that NeoSTX attenuated clinical signs of joint inflammation relative to saline-treated controls.

### 3.2. Synovial Fluid Cytology

SF obtained from NeoSTX-treated joints maintained normal color and viscosity, with no signs of hemorrhage. In contrast, saline-treated joints tended to exhibit a red hue and decreased viscosity. SF collected from TA-treated horses showed reddish-brown color, suggestive of hemarthrosis, and decreased viscosity.

**Figure 3:**
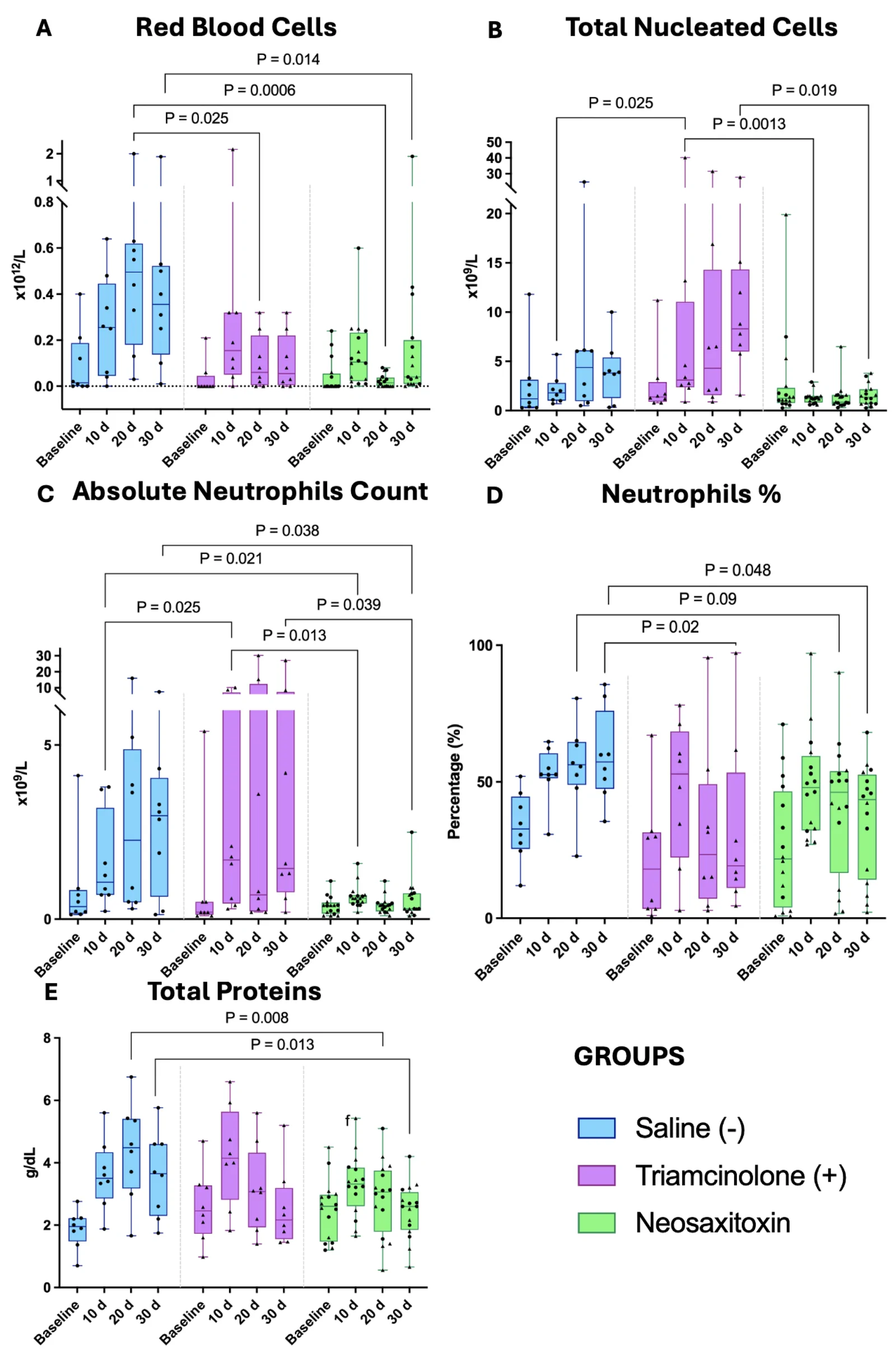
Synovial fluid cellular composition. Boxplots (median, IQR, range) for RBC (**A**), TNCC (**B**), neutrophils (**C**), neutrophils % (**D**), and total proteins (**E**) at baseline and days 10, 20, and 30. NeoSTX maintained lower RBC and neutrophil counts compared with saline and triamcinolone. Triamcinolone induced transient TNCC elevations and a mononuclear cell increase at day 30, while saline controls exhibited sustained RBC and neutrophil increases. (▲) represents individual data points from the NeoSTX vs. TA comparison, and (●) represents individual data points from the NeoSTX vs. saline comparison.

Red blood cell (RBC) counts were comparable across groups at baseline (pre-model induction). By days 20 and 30, NeoSTX-treated joints, showed lower RBC compared with saline-treated joints (Figure 3A). Total nucleated cell (TNCC) counts were also similar across groups at baseline. However, from day 10 onward, joints treated with TA displayed a significant increase in TNCC at days 10 (*p* = 0.0013) and 30 (*p* = 0.019) (Figure 3B).

Neutrophil proportions were increased in all groups on day 10, consistent with an acute inflammatory response to the model induction. Notoriously, in NeoSTX-treated joints neutrophil counts significantly decreased towards baseline when compared to both saline- and TA-treated joints (Figure 3C,D). Mononuclear cell dynamics further depicted a time-dependent effect of NeoSTX, in which NeoSTX-treated joints showed a progressive recovery of baseline levels of mononuclear cells, yet these findings failed to reach significance. TA-treated joints showed a progressive increase in mononuclear cells concentration; not observed in saline-treated joints (Figure S2).

In response to the creation of the model there was an increase in synovial fluid TP in all groups at 10 days. Nonetheless, by days 20 and 30, TA- and NeoSTX-treated joints exhibited a progressive decrease in TP concentrations that were only significant for NeoSTX compared to saline-treated joints (*p* = 0.0008 and 0.013, respectively) (Figure 3E).

### 3.3. Synovial Fluid Cytokines and Chemokines

**Figure 4:**
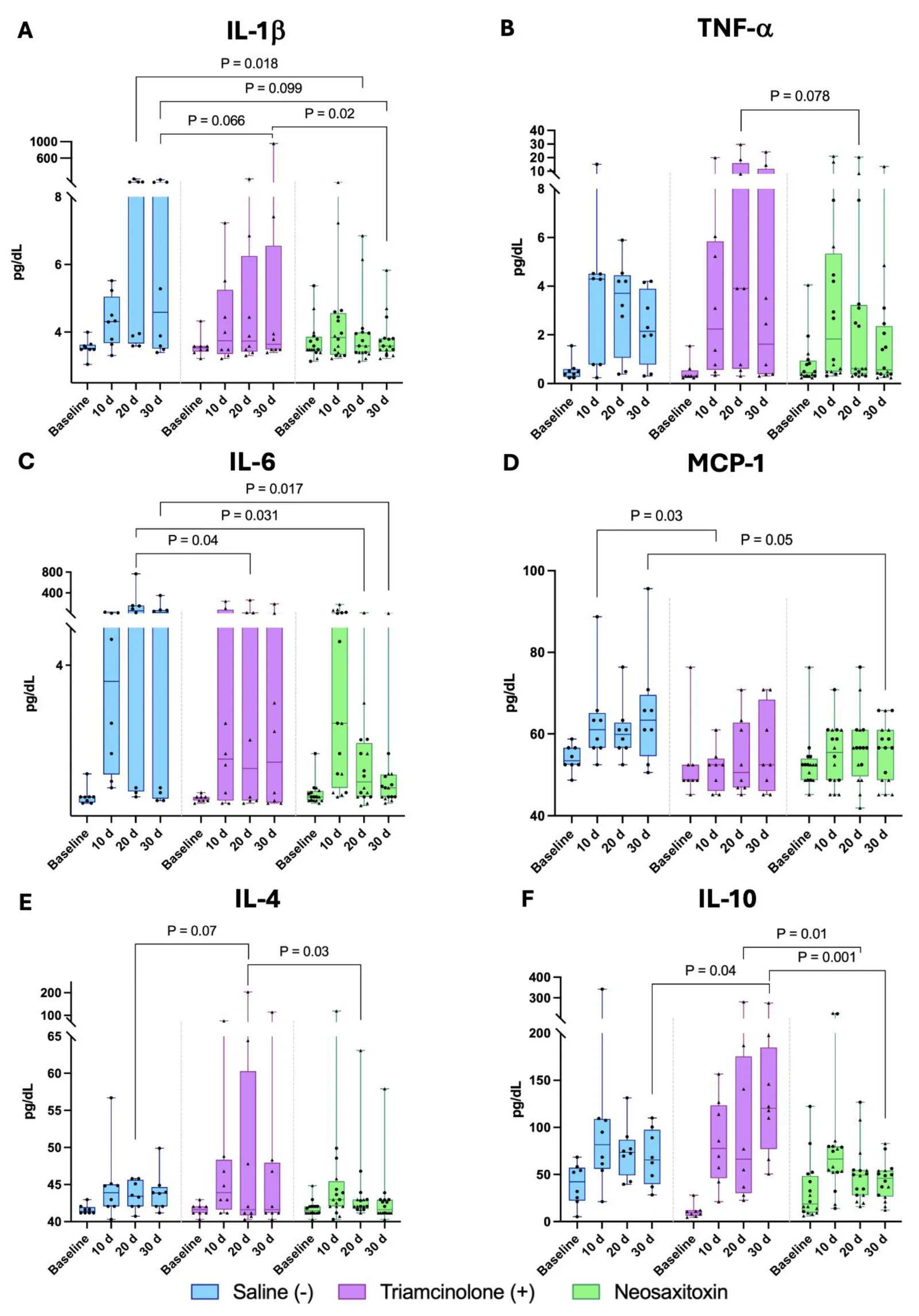
Box and whiskers plots of synovial fluid concentrations of cytokines/chemokines exhibiting significant differences between groups over the course of the study. Boxes representing the 25th and 75th interquartile percentile and median values, with whiskers representing the range of changes. Joints treated with NeoSTX exhibited lower concentrations of the acute inflammatory mediators (**A**) IL-1β, (**B**) TNF-α, (**C**) IL-6 and (**D**) MCP-1 than saline-treated controls. On the other hand, NeoSTX-treated joints exhibited lower concentrations of the pro-resolving mediators (**E**) IL-4 and (**F**) IL-10 than TA-treated controls. (▲) represents individual data points from the NeoSTX vs. TA comparison, and (●) represents individual data points from the NeoSTX vs. saline comparison.

NeoSTX-treated joints exhibited lower concentrations of both pro-inflammatory cytokines and chemokines (IL-1β, IL-6, TNF-α, and MCP-1) (Figure 4A–D) (IFN-γ, IL-2, IP-10, and IL-18, Figure S2) compared with TA- and saline-treated controls. Concentrations of IL-1β in NeoSTX-treated joints were lower compared with saline group at Day 20 (*p* = 0.018), and exhibited a trend at Day 30 (*p* = 0.099). These results were comparable to those observed in the TA-treated group, which also showed a trend toward lower IL-1β concentrations at day 30 relative to the saline-treated joints. Notably, direct comparison between the NeoSTX and TA groups revealed significantly lower IL-1β concentrations in the NeoSTX-treated joints at day 30 (*p* = 0.02) (Figure 4A). A similar pattern was observed for IL-6 and TNF-α where NeoSTX-treated joints exhibited earlier recovery towards baseline concentrations. NeoSTX-treated joints exhibited a trend for lower TNF-α concentrations than TA-treated joints at Day 20 (*p* = 0.078) (Figure 4B), and a lower IL-6 concentration than saline-treated joints on days 20 and 30 (Figure 4C).

Other pro-inflammatory cytokines, including IFN-γ and IL-2, were also upregulated in the saline- and TA-treated groups when compared with NeoSTX-treated joints, in which their concentrations were near-baseline levels (Figure S2A,B). A similar pattern was observed for other cytokines associated with OA, such as IP-10 and IL-18, which showed significantly lower concentrations on days 20 and 30 in the NeoSTX-treated group when compared to saline-treated controls (Figure S2C,D). Following model induction, MCP-1 concentrations increased in all three groups. NeoSTX-treated joints were the only ones to exhibit a decrease in MCP-1 concentrations, which were ultimately significantly lower than saline-treated controls at day 30 (*p* = 0.05) (Figure 4D).

Regulatory cytokines (IL-4, IL-10) remained at low concentrations in the NeoSTX-treated group compared with the saline- and TA-treated groups. IL-10 concentrations peaked at day 10 following model induction. While it progressively increased in TA treated joints, it progressively decreased in the NeoSTX-treated group, significantly differing from TA joints at Days 20 and 30 (Figure 4E,F). Overall, NeoSTX-treated joints maintained a cytokine profile near-baseline, hence without inducing compensatory activation of anti-inflammatory pathways following acute inflammation.

Finally, estimated marginal means analysis revealed time-dependent differences across inflammatory mediators (Table S1). NeoSTX-treated joints exhibited smaller effect sizes. In contrast, saline-treated joints showed a progressive increase in pro-inflammatory cytokines and chemokines, with several comparisons versus NeoSTX yielding significant at 95% confidence intervals. TA-treated joints displayed a more variable response, with some differences relative to NeoSTX- and saline-treated joints.

### 3.4. Cartilage and Synovial Membrane Evaluation

#### 3.4.1. Gross Pathology

**Figure 5:**
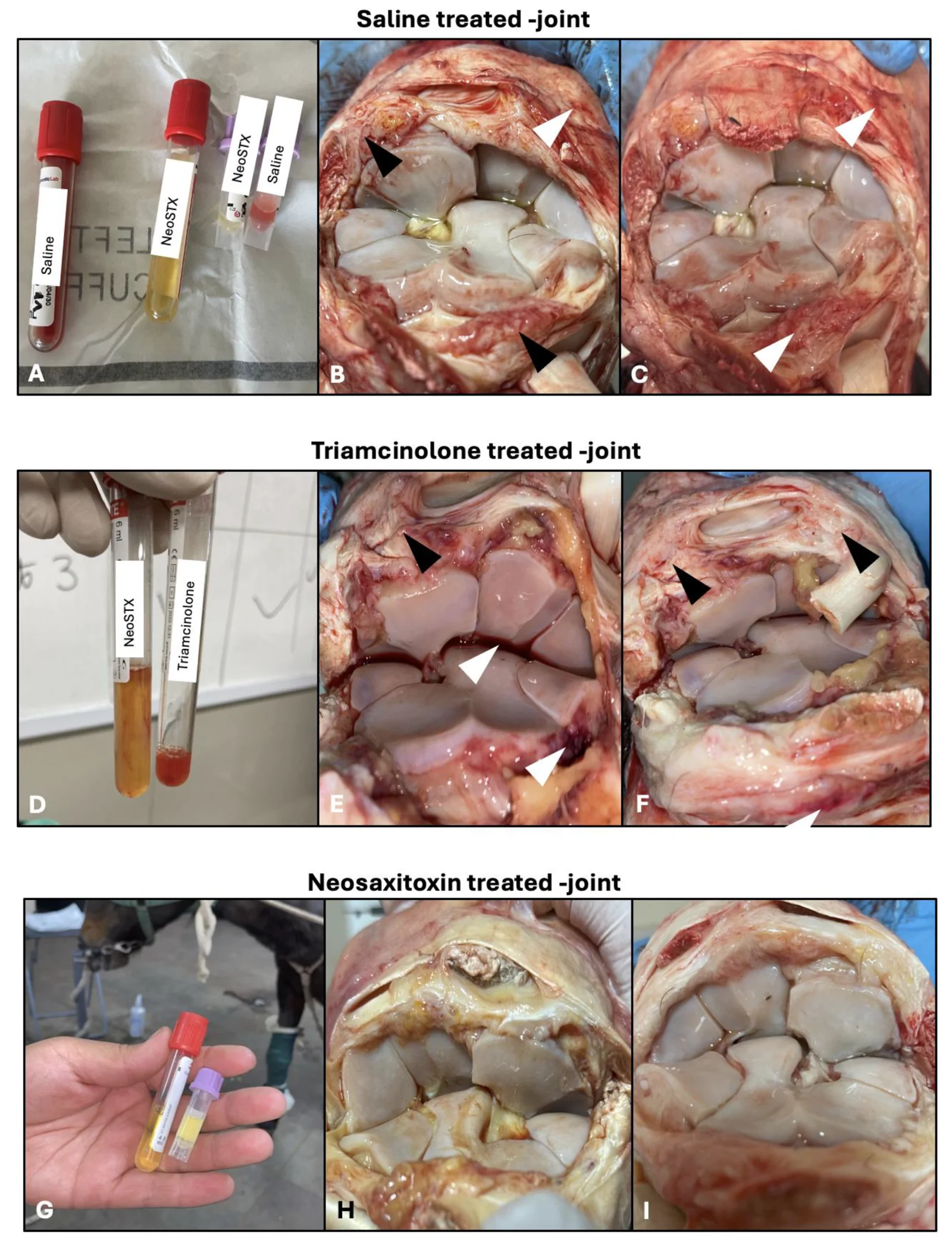
(**A**) Synovial fluid collection and comparison between Saline- and NeoSTX-treated joints. (**B**,**C**) Depicting macroscopically inflamed synovium characterized by swelling (black arrowheads) and periarticular and intra-articular hemorrhage (white arrowheads) in joints treated with saline and (**E**,**F**) joint treated with TA. (**D**) Comparison synovial fluid gross appearance between the TA- and NeoSTX-treated joints. There is some blood contamination in the NeoSTX sample, probably due to blood contamination during the procedure. Observe the reddish and scarce amount of synovial fluid in the Triamcinolone group, this outcome was constantly observed in the Triamcinolone group after repeated treatment administration. (**G**) Synovial fluid collection of NeoSTX-treated joint, showing normal macroscopic characteristics. (**H**,**I**) Representative (subjects 8 and 12) postmortem middle carpal joint images of NeoSTX-treated joints characterized by less swelling in the synovium and peri-articular and intra-articular hemorrhage. No major changes were evidenced in the cartilage surfaces.

Gross inspection of saline-treated joints, consistently evidenced a mildly swollen and hyperemic synovial membrane characteristic of chronic inflammation (Figure 5B,C). TA-treated joints display marked macroscopic changes, including synovial hyperemia, cartilage surface irregularities, and hemorrhagic synovial fluid (Figure 5D). TA-treated joints also exhibited a more pronounced synovial membrane hyperemia and thickening than saline or NeoSTX, with pannus-like tissue (Figure 5E,F). In contrast, NeoSTX-treated joints revealed smoother, glistening articular cartilage surfaces, with uniform coloration and no visible fibrillation or erosions. The synovium appeared pale pink and thin, with minor villous hypertrophy and hyperemia. No evidence of exudate, hemorrhage, or pannus formation was observed (Figure 5H,I).

#### 3.4.2. Synovial Membrane Histology

**Figure 6:**
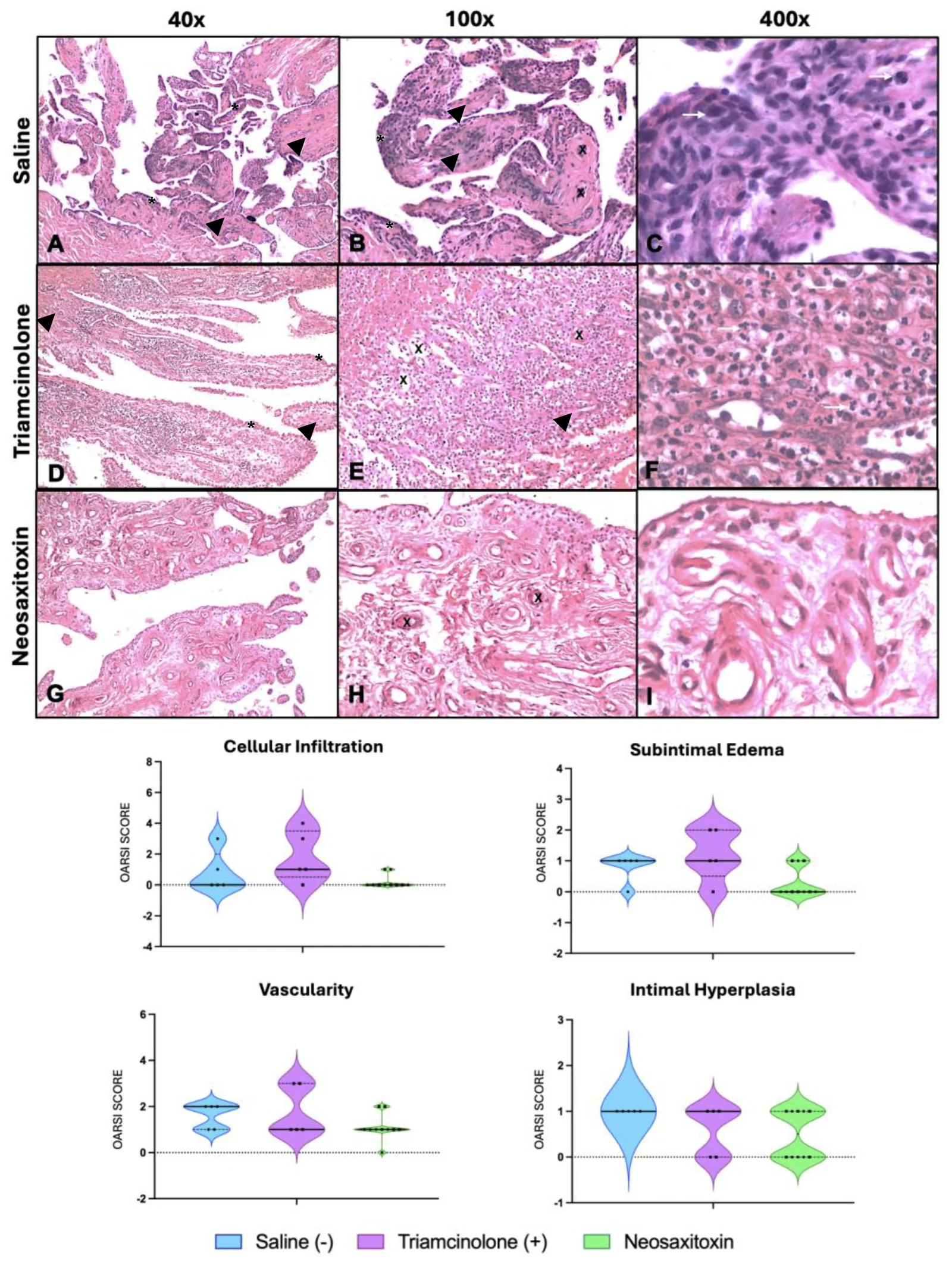
Representative hematoxylin and eosin images of the synovial membrane depicting differences in histologic parameters between saline- (**A**–**C**), Triamcinolone- (**D**–**F**), and NeoSTX-treated (**G**–**I**) joints after 30 days of OA induction and treatment administration. NeoSTX-treated joints exhibited lower scores for all histologic aspects of inflammation, although these differences were not statistically significant. Hyperplasia of the intimal layer (*) in (**A**,**D**) (40×), and fibrosis of the subintimal collagen fibers (black arrow heads) in (**A**,**D**) (40×), (**B**,**E**) (100×). Prescence of vascularization (X) in (**B**,**E**,**H**), 100×. Increase in inflammatory cellular infiltration in Triamcinolone- and Saline-treated synovial membrane (white arrows) in (**C**,**F**), 400×. Violin plots of OARSI score between groups. Doted lines within the violin plots representing the 25th and 75th interquartile percentile and median values. (■) represents individual data points from the NeoSTX vs. TA comparison, and (●) represents individual data points from the NeoSTX vs. saline comparison. OARSI, Osteoarthritis Research Society International.

Saline-treated joints also showed abnormal synovial membrane histology characterized by mild intimal hyperplasia (2–4 layers) and slight congestion of synovial villi (Figure 6A). Mild inflammatory infiltrate in subintimal layers was also evidenced, but less intense than TA (Figure 6B), accompanied by occasional inflammatory cells (Figure 6C).

Opposingly, TA-treated joints showed increased intimal hyperplasia characterized by 4–5 rows of intimal cells, with focal, mild increase in vascularity (Figure 6D). TA-treated joints also exhibited moderate cellular infiltration in the subintimal layer (Figure 6E,F) and exhibited the highest overall OARSI scores for cellular infiltration, intimal hyperplasia, as well as the composite score.

Neosaxitoxin-treated synovium displayed a thin intimal lining, typically 1–2 cell layers, with minimal hyperplasia, inflammatory cells, or subintimal edema (Figure 6G). The subintimal layer was loosely organized with occasional small areas of cell infiltration and focal, mildly increased vascularity (Figure 6H). Slight subintimal fibrosis was evidenced throughout the sections (Figure 6I). Additionally, all individual OARSI scores were low, near baseline.

#### 3.4.3. Cartilage and Subchondral Bone Histology

**Figure 7:**
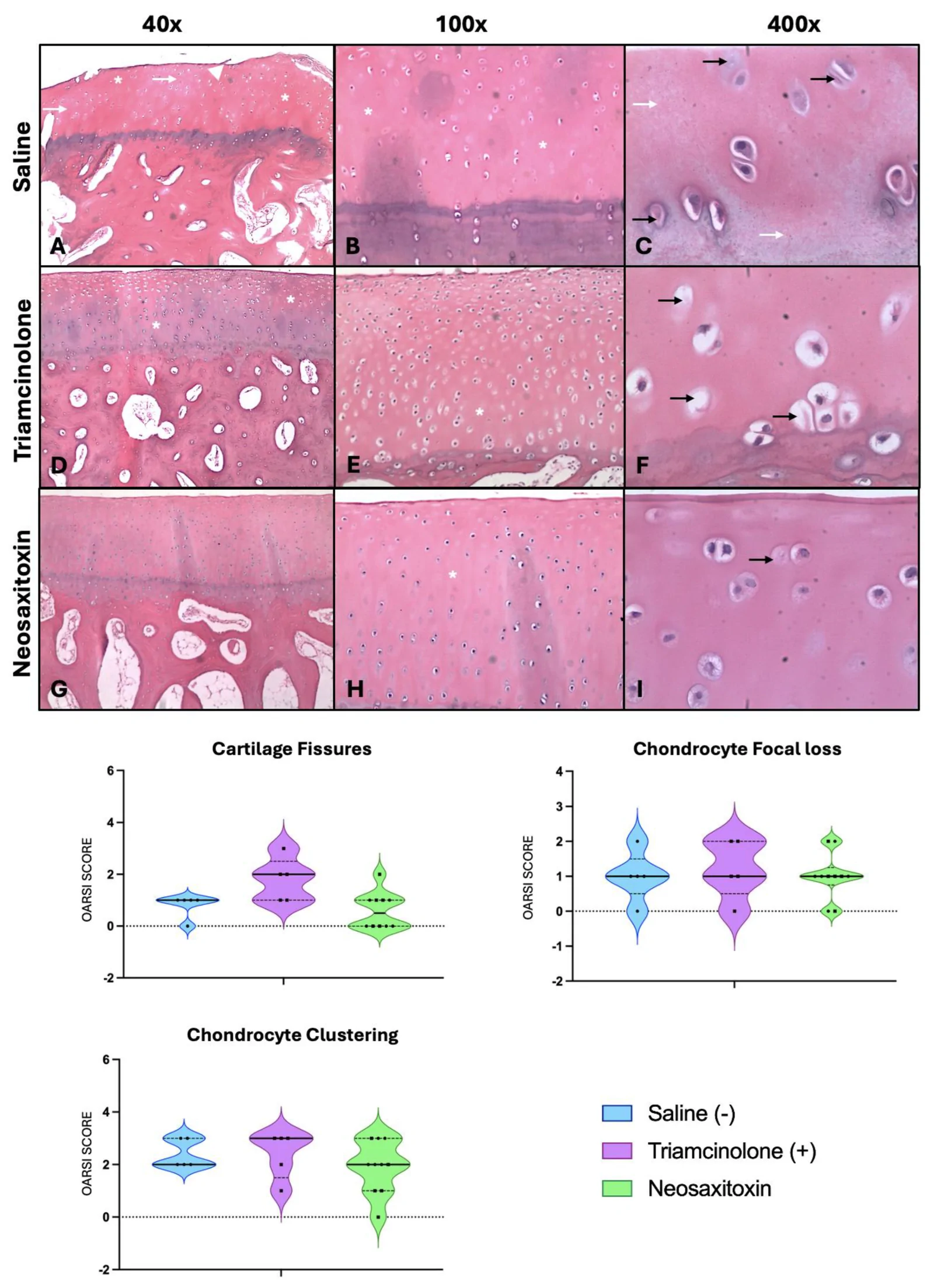
Representative hematoxylin and eosin images of hyaline cartilage depicting differences in histologic parameters between saline- (**A**–**C**), Triamcinolone- (**D**–**F**), and NeoSTX-treated (**G**–**I**) joints after 30 days of OA induction (carpal chip model) and treatment administration. NeoSTX-treated joints exhibited lower scores for all histologic aspects of cartilage degradation, although these differences were not statistically significant. Cartilage fissures (white arrowheads) and higher chondrocyte focal loss (*) were observed in triamcinolone- and saline-treated joints, (**A**,**D**), 40× and (**B**,**E**), 100×. Chondrocyte necrosis near the articular surface is more evident in the Triamcinolone and Saline groups (black arrows), as shown in (**C**,**F**,**I**) (400×). Additionally, decreased H&E staining uptake was observed primarily in saline-treated horses (white arrows), as shown in (**A**,**C**). Violin plots of OARSI score between groups. Doted lines within the violin plots representing the 25th and 75th interquartile percentile and median values. (■) represents individual data points from the NeoSTX vs. TA comparison, and (●) represents individual data points from the NeoSTX vs. saline comparison. OARSI, Osteoarthritis Research Society International.

Articular cartilage from the saline-treated joint displayed mild superficial layer detachment and occasional fissures (Figure 7A). Chondron formation (cluster of clonal chondrocytes) was also observed, and doublets and triplets were identified along superficial aspect of the articular cartilage. Matrix staining was slightly uneven compared to NeoSTX (Figure 7B) with occasional degenerative chondrocytes (Figure 7C).

TA-treated joints showed more pronounced surface irregularities and fissuring that extended into the middle zone. Chondron formation was more evident and characterized by 2–3 chondrocytes (doublets & triplets) along the superficial zone. The subchondral bone displayed prominent vascular channels (Figure 7D), and some fissures or clefts extend through the tidemark (Figure 7E). Moreover, nuclear pyknosis and cytoplasmic condensation were more often observed, suggesting higher focal cell loss (Figure 7F).

NeoSTX-treated group showed slight fibrillation which was restricted to surface and superficial zone. There was also homogeneous chondrocyte distribution, with slight chondrocyte clustering (doublets) along the superficial layer. NeoSTX-treated joints had minor disruption of the calcified cartilage affecting less than 1–2 mm of the subchondral bone (Figure 7G). Matrix staining was uniform (Figure 7H). At higher magnification, chondrocytes showed normal morphology, with well-defined lacunae and no evidence of necrosis or pyknosis, with occasionally focal loss (Figure 7I).

**Figure 8:**
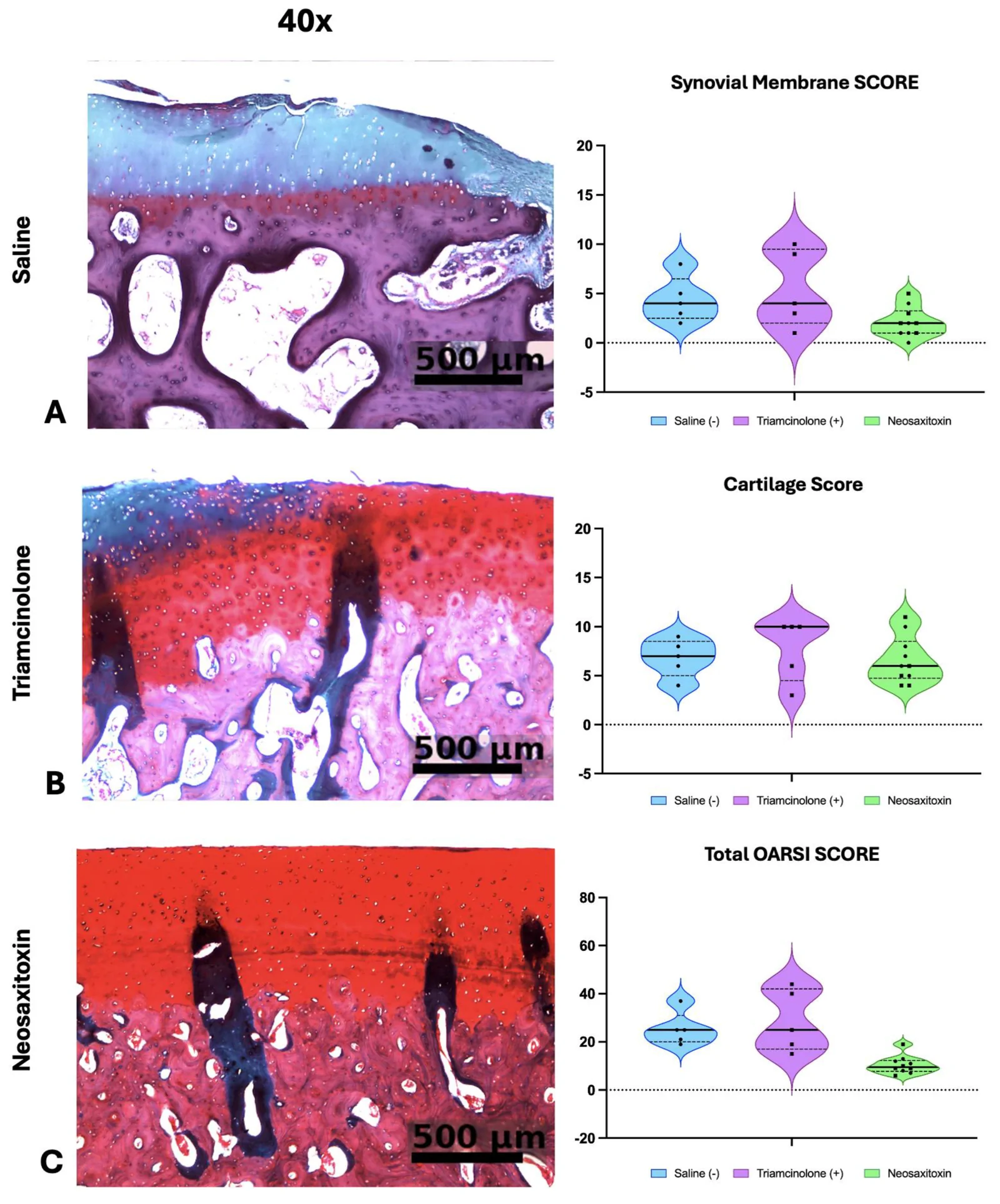
Representative Safranin-O images of hyaline cartilage and subchondral bone depicting differences in histologic parameters between saline- (**A**), Triamcinolone- (**B**), and NeoSTX-treated (**C**) joints after 30 days of OA induction and treatment administration. Cartilage from joints treated with NeoSTX exhibited preserved proteoglycan content, depicted by red staining, while those treated with Triamcinolone showed proteoglycan loss from the superficial layer of the cartilage, and horses treated with saline showed significantly proteoglycan loss throughout the cartilage. Violin plots of OARSI score between groups. Doted lines within the violin plots representing the 25th and 75th interquartile percentile and median values. (■) represents individual data points from the NeoSTX vs. TA comparison, and (●) represents individual data points from the NeoSTX vs. saline comparison. OARSI, Osteoarthritis Research Society International.

Finally, safranin-O staining of cartilage samples from joints treated with saline- showed proteoglycan content loss (50–75%) throughout the cartilage layers (Figure 8A) while those treated with TA showed a mild-significant proteoglycan loss at the superficial layer of the cartilage (25–50%, Figure 8B). NeoSTX exhibited overall a more preserved or a mild (<25%) loss of proteoglycan content (Figure 8C), In summary, NeoSTX-treated joints exhibited the lowest OARSI cartilage score assessment between groups with higher proteoglycan content preservation.

## 4. Discussion

This study investigated the effects of intra-articular NeoSTX on synovial inflammation and joint tissue homeostasis in an experimentally induced model of equine OA. Treatment with NeoSTX was associated with modulation of the synovial inflammatory response, reflected by improvements in clinical, cytological and histopathological markers of inflammation that were associated with lower concentrations of IL-1β, IL-6, MCP-1 in synovial fluid and cartilage preservation. This is the first controlled, in vivo study to evaluate the potential effects of NeoSTX as therapy for osteoarthritis, showing that selective NaV channel blockade modulates synovial inflammation and recovery of joint homeostasis. These findings extend our previous work demonstrating the feasibility of intra-articular NeoSTX administration in healthy joints [45], further supporting continued investigation in this field.

Lower concentrations of the pro-inflammatory cytokines and chemokines (IL-1β, IL-6, and MCP-1) in NeoSTX-treated joints are comparable to our previous in vitro study where blocking voltage-gated sodium channels (NaVs) in macrophages reduced the expression of several pro-inflammatory mediators, including IL-1β, IL-6, TNF-α, and inducible nitric oxide synthase (iNOS) [33]. Furthermore, the effect of NeoSTX on cytokine/chemokine concentrations in our in vivo study is also further supported by similar reports where saline-treated joints sustained higher concentrations of inflammatory mediators [48,50,51]. In contrast, while TA-treated joints experienced an anti-inflammatory response, some of the differences observed between NeoSTX and TA may be explained by the different mechanisms of action between the two molecules, which warrant further research to unveil the molecular mechanisms by which NeoSTX better preserved joint tissues and recovered joint homeostasis [19,51,52,53].

Pro-resolving cytokines such as IL-4 and IL-10 are known to resolve inflammation and are expected to have increased expression during inflammation resolution [7,54]. Nonetheless, such increases are directly proportional to the expression of so-called pro-inflammatory cytokines such as TNF-α [55,56] following disruption of homeostasis. In NeoSTX-treated joints, their levels returned closer to baseline after treatment, whereas TA-treated joints exhibited sustained elevation. Although these cytokines are known for their pro-resolving and anabolic effect during tissue repair, sustained IL-10 expression in TA-treated joints in our study depicts the difference between anti-inflammation and inflammation resolution [7,57]. Inflammation resolution is an active process mediated by a balance of pro-inflammatory and pro-resolving mediators that drive tissue repair and recovery of homeostasis. A key determinant of successful inflammation resolution is the acute inflammatory response itself, which serves as a critical trigger for activating pro-resolving pathways [55,56]. Blockage of inflammatory mediators with anti-inflammatory drugs block tissue repair responses, which generate delayed or sustained waves of both pro-inflammatory and pro-resolving mechanism as recovery of homeostasis has not been achieved [7,58]. This is reflected by the sustained concentrations of TNF-α, IL-6, IL-10 IL-2 and IFN-γ in TA-treated joints in our study as a normal physiologic response to attempt to restore homeostasis. In contrast, the lower cytokine concentrations in NeoSTX-treated horses can be explained by increased tissue resistance to inflammatory stimulus [8]. These observations are further supported by clearly different synovial fluid and histological outcomes from NeoSTX-treated joints exhibiting superior overall joint tissue preservation. Analysis of synovial fluid cellularity also revealed lower red blood cell and neutrophil counts in NeoSTX-treated joints compared with saline-treated joints. These cellular responses are consistent with the cytokine and chemokine profiles observed in each treatment group. The cellular response observed in NeoSTX-treated joints is consistent with previous studies demonstrating that regulation of synovial cellularity is essential for restoring joint homeostasis following inflammatory injury [7].

Lower OARSI scores for the synovium and osteochondral units of NeoSTX-treated joints interpreted in the lights of the combined outcome measures suggest mitigation of synovial inflammation and better preservation of joint homeostasis. On the other hand, TA- and saline-treated joints exhibited greater proteoglycan loss, consistent with their respective clinical, cytological, and inflammatory mediator profiles. Although TA-treated joints showed less proteoglycan loss (Safranin O staining) than saline-treated joints, these results should be interpreted alongside the concomitant synovial alterations observed in TA-treated joints, highlighting the complex and tissue-specific effects of intra-articular corticosteroids [52,53,58,59,60,61,62]. While TA remains the primary drug for short-term control of joint inflammation, its potential deleterious effects on joint tissues over time must be carefully considered when designing therapeutic strategies.

Treatment with NeoSTX also resulted in significant improvements in joint flexion range, circumference, and temperature compared to saline-treated joints, all well characterized indicators of joint inflammation. NeoSTX results were comparable to those observed in the TA-treated joints. While TA is known to yield marked short-term anti-inflammatory effects [43,49,59,60], TA-treated joints evidenced higher concentration of pro-inflammatory cytokines (IL-1β, TNF-α, IFN-γ and IL-18) during the final stages of the study, likely contributing to the ultimately worst histopathological changes observed in TA-treated joints.

**Figure 9:**
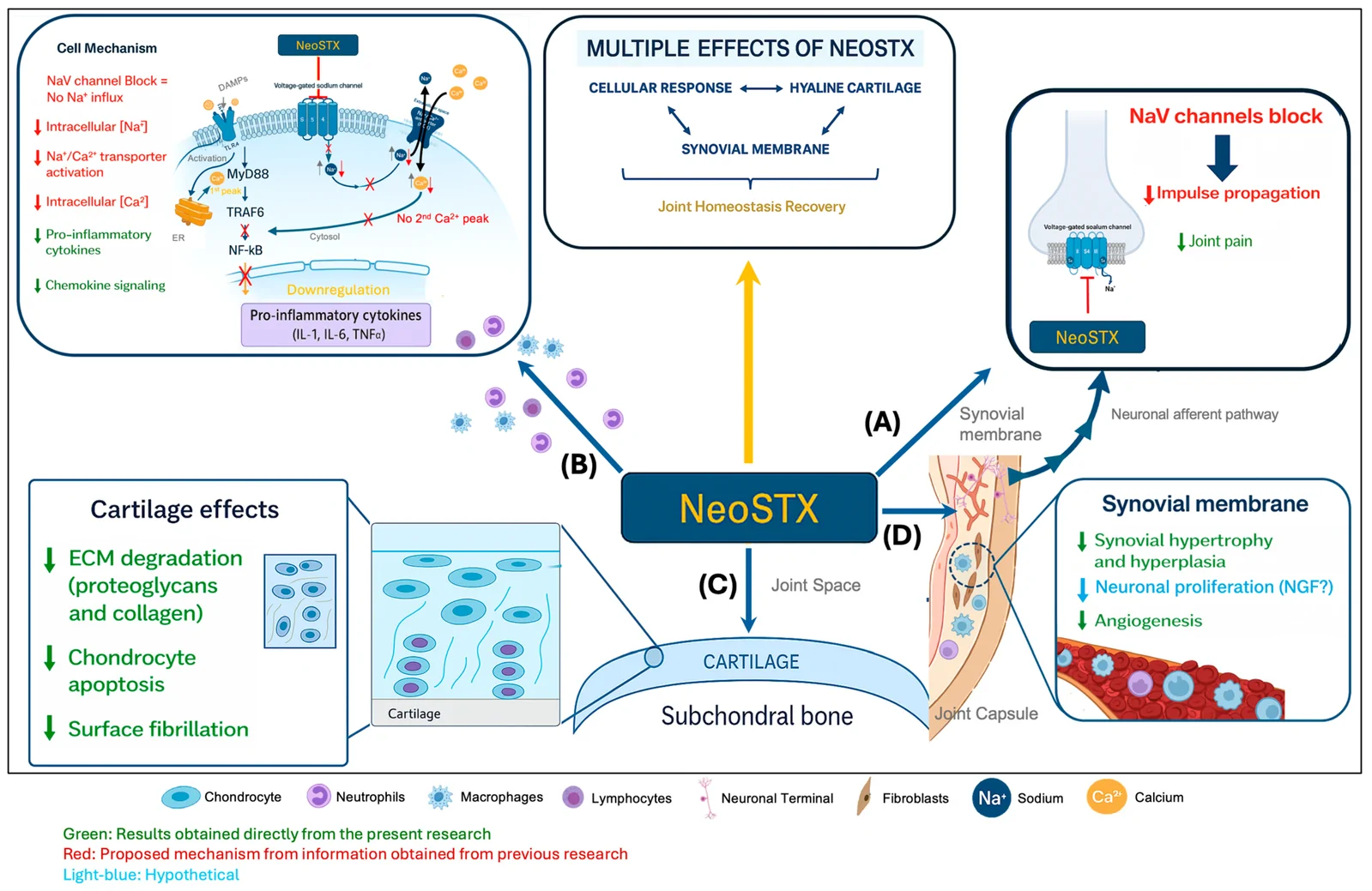
Proposed mechanistic hypothesis underlying the effects of intra-articular administration of NeoSTX. (**A**) NeoSTX blocks voltage-gated sodium channels (NaV), reducing neuronal impulse propagation and joint pain. (**B**) At the cellular level, blockade of Na^+^ influx and Na^+^/Ca^2+^ transport downregulates intracellular calcium signaling, thereby inhibiting NF-κB activation and reducing the release of pro-inflammatory cytokines (IL-1, IL-6) and chemokine signaling. (**C**) In cartilage, NeoSTX decreases extracellular matrix degradation (proteoglycans and collagen), limits chondrocyte apoptosis, and reduces fibrillation. (**D**) In the synovial membrane, NeoSTX attenuates hypertrophy, hyperplasia, angiogenesis, and possibly neuronal proliferation (related to NGF). Collectively, these effects contribute to the recovery of joint homeostasis by modulating cellular responses in cartilage and synovium. Red: Previous research for proposed mechanism [23,27,31,33,34,75,76].

Our combined findings support the hypothesis that NeoSTX exerts a broad homeostatic effect, rather than blocking inflammatory pathways required for recovery of homeostasis. The mechanism through which NeoSTX exerts its homeostatic effects remains hypothetical but previous reports and the results of this study suggest that voltage-gated sodium channels (NaV) in macrophages and chondrocytes [63] are involved in the pathophysiology of several inflammatory and degenerative diseases [32,64]. This concept has recently gained attention in OA research, where NaV channels have been identified as potential biomarkers and therapeutic targets due to their involvement in inflammatory signaling and cellular activation [65,66,67]. Therefore, aligned with our findings, these early observations further substantiate our hypothesis in which NeoSTX-mediated NaV blockade prevents the initial sodium influx, thereby inhibiting the secondary calcium peak required for downstream signal transduction [31]. A proposed mechanism is summarized in Figure 9. Although the proposed mechanism provides a biologically plausible explanation for the observed findings, future studies are required to define the homeostatic nature of the response to NeoSTX.

The modified carpal chip model employed in this study provided a robust and reproducible platform for the induction of osteoarthritis-associated synovial inflammation in horses. Although traditional protocols incorporate high-speed galloping to exacerbate joint loading, accumulating evidence indicates that controlled, repetitive mechanical stimulation applied to an injured joint is enough to trigger the inflammatory cascade and structural changes characteristic of early OA [39,43,68]. In the present study, the adapted exercise protocol yielded consistent clinical, cytological, and molecular variations, including joint effusion and temperature, inflammatory biomarker profiles and histology confirming successful model induction.

Despite these promising results, our sample size was limited, even though it reproduced that of studies of a similar nature [40,42,43]. Another limitation in our study is that intra-articular triamcinolone acetonide undergoes some degree of systemic absorption [69,70,71,72,73] and, therefore, could have introduced some bias to our interpretation of the effects of NeoSTX in joints of horses also receiving TA. However, our assessment of the data from the NeoSTX joints from both treatment (TA) and control (saline) groups, combined or separated, showed that the outcomes from NeoSTX joints did not differ between groups for several parameters (Table S2), suggesting that TA effects were localized to the treated joint [74]. Another limitation of this study is the relatively short 30-day follow-up period, which precluded assessment of the long-term effects of NeoSTX. The latter combined with the incomplete histopathological sampling reduced the sample size available for tissue analyses. Yet, no imputation of missing data was performed, and analyses were, therefore, conducted using complete-case analysis on the available samples.

## 5. Conclusions

The combined interpretation of our findings supports targeting NaV channels through repeated NeoSTX joint injections as a promising therapy for synovial inflammation in early stages of OA. Compared with corticosteroids, NeoSTX may offer potential advantages in long-term joint preservation by combining homeostatic immunomodulatory effects. However, the long-term effects of intra-articular NeoSTX on joint preservation and osteoarthritis progression require further investigation. Collectively, these findings warrant further investigation on NaV channel blockage as a promising immunomodulatory strategy for restoring synovial homeostasis in osteoarthritic joints.

## Data Availability

The original contributions presented in this study are included in the article/Supplementary Material. Further inquiries can be directed to the corresponding author.

## Supplementary Materials

The following supporting information can be downloaded at: https://www.mdpi.com/article/10.3390/biom16081142/s1, Figure S1. Radiographic examination at day 30 post model induction. Images obtained from the right forelimb (RF) and left forelimb (LF) from subject 8. Dorso lateral – palmaro medial oblique view (DLPMO) (**A**) and dorso palmar view (DP) (**B**) of NeoSTX-treated joints. DLPMO (**C**) and DP (**D**) of TA-treated joints. Figure S2. Box and whiskers plots of synovial fluid concentrations of cytokines/chemokines exhibiting significant differences between groups over the course of the study. Boxes representing the 25th and 75th interquartile percentile and median values, with whiskers representing the range of changes. Joints treated with NeoSTX exhibited lower concentrations of inflammatory mediators (**A**) IFN-gamma, (**B**) IL-2, (**C**) IP-10, and (**D**) IL-18 than saline-treated controls. On the other hand, NeoSTX-treated joints exhibited lower concentrations of (**A**) IFN-gamma, (**B**) IL-2, and (**D**) IL-18 than TA-treated controls at day 20. No differences were observed in Mononuclear cell count (**E**). (▲) represents individual data points from the NeoSTX vs. TA comparison, and (●) represents individual data points from the NeoSTX vs. saline comparison. Table S1. Estimated marginal mean differences for synovial fluid mediators comparing saline and triamcinolone treatments relative to NeoSTX at each time point are presented with corresponding 95% confidence intervals (CI). Positive and negative values indicate higher or lower concentrations relative to NeoSTX, respectively. Statistical significance was defined as 95% CI not crossing zero. Time points are shown relative to osteochondral fragment induction. Table showing only parameters and time points were differences were observed. Table S2. Multiple between-group comparisons demonstrating the comparable behavior of both NeoSTX-treated joints throughout the study, together with comparisons between the TA- and saline- treated joints, including corresponding significant *p*-values.

## Abbreviations

The following abbreviations are used in this manuscript:

CBC	Complete blood count
EMMs	Estimated marginal means
GLMM	Generalized linear mixed model
IA	Intraarticular
LLOD	Lower limit of detection
NaV	Voltage-gated sodium channel
NeoSTX	Neosaxitoxin
OA	Osteoarthritis
OARSI	Osteoarthritis Research Society International
PPARG	Peroxisome proliferator-activated receptor gamma
RBC	Red blood cells
RCB	Radial carpal bone
SF	Synovial fluid
TA	Triamcinolone
TNCC	Total nucleated cells
TP	Total proteins
