# Supplementary material for: Neosaxitoxin Downregulates Inflammation in an Equine In Vivo Model of Osteoarthritis
Source: Biomolecules. 2026 Aug 6;16(8):1142. doi: 10.3390/biom16081142 (PMC13511278; doi:10.3390/biom16081142)
Supplement: Supplementary file 1 [file biomolecules-16-01142-s001.zip › biomolecules-4381135-supplementary.pdf]

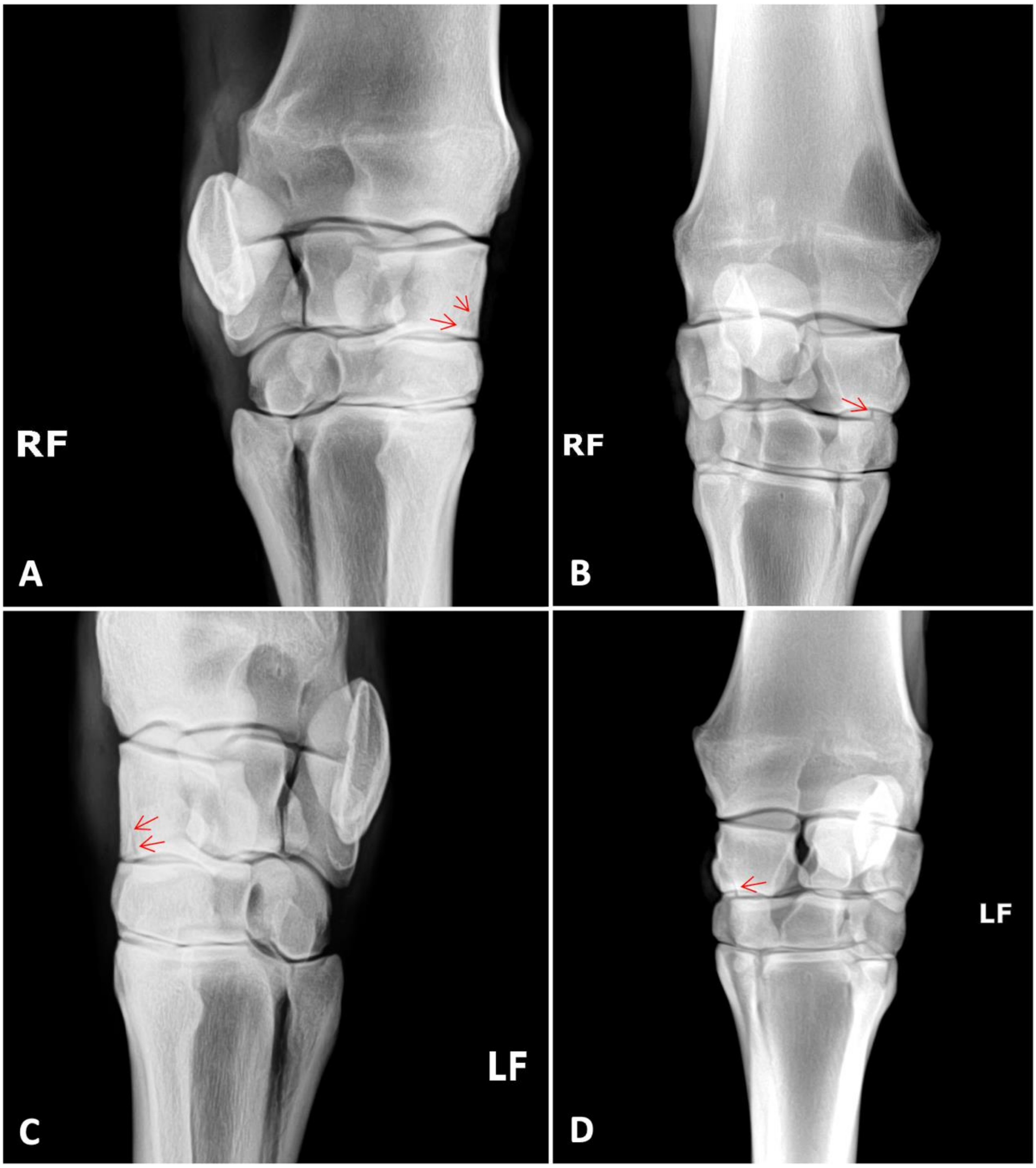

Figure S1. Radiographic examination at day 30 post model induction. Images obtained from the right forelimb (RF) and left forelimb (LF) from subject 8. Dorso lateral – palmaro medial oblique view (DLPMO) (**A**) and dorso palmar view (DP) (**B**) of NeoSTX-treated joints. DLPMO (**C**) and DP (**D**) of TA-treated joints.

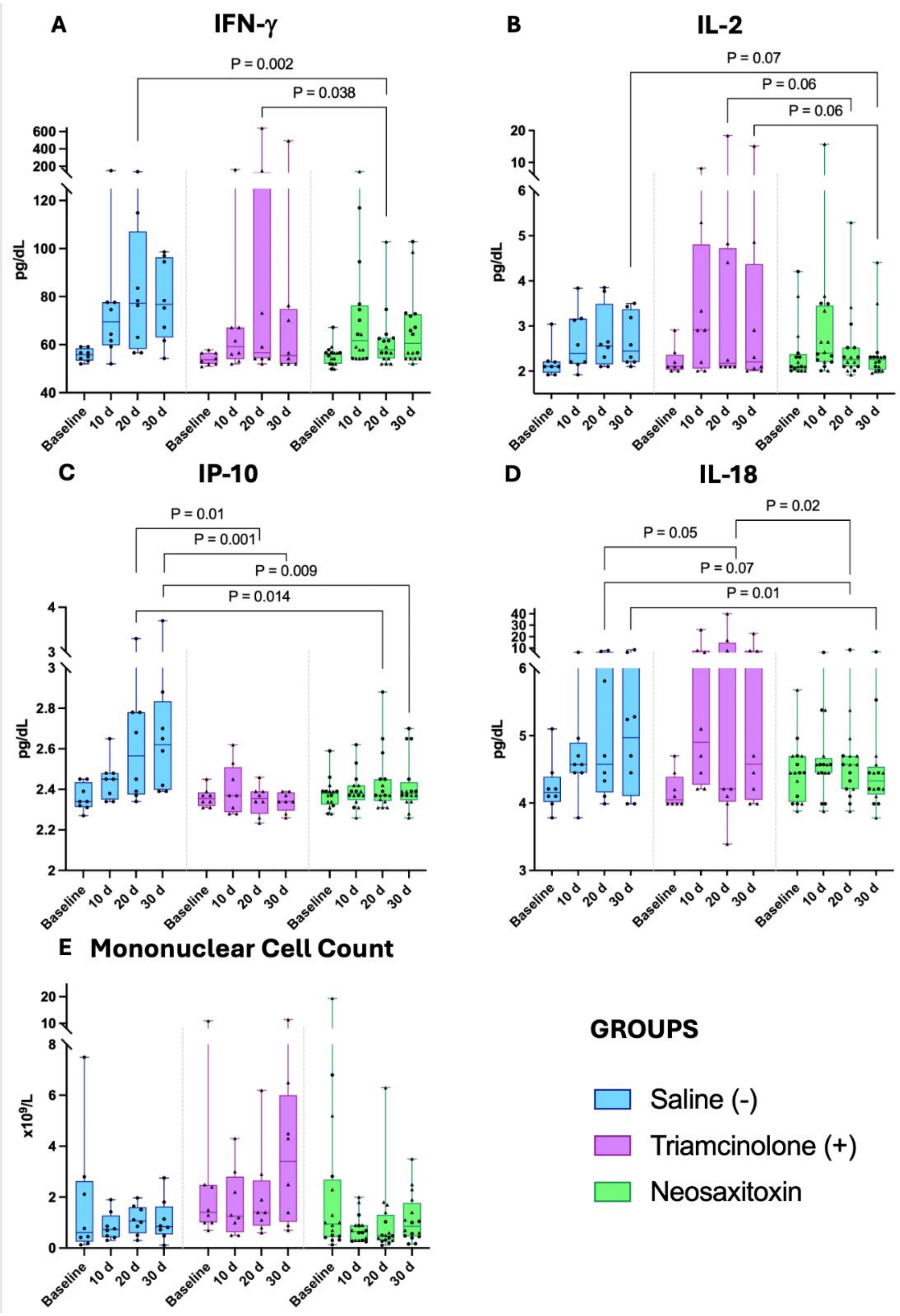

Figure S2. Box and whiskers plots of synovial fluid concentrations of cytokines/chemokines exhibiting significant differences between groups over the course of the study. Boxes representing the 25th and 75th interquartile percentile and median values, with whiskers representing the range of changes. Joints treated with NeoSTX exhibited lower concentrations of inflammatory mediators **(A)** IFN-gamma, **(B)** IL-2, **(C)** IP-10, and **(D)** IL-18 than saline-treated controls. On the other hand, NeoSTX-treated joints exhibited lower concentrations of **(A)** IFN-gamma, **(B)** IL-2, and **(D)** IL-18 than TA-treated controls at day 20. No differences were observed in Mononuclear cell count (**E**). (▲) represents individual data points from the NeoSTX vs. TA comparison, and (●) represents individual data points from the NeoSTX vs. saline comparison.

Table S1. Estimated marginal mean differences for synovial fluid mediators comparing saline and triamcinolone treatments relative to NeoSTX at each time point are presented with corresponding 95% confidence intervals (CI). Positive and negative values indicate higher or lower concentrations relative to NeoSTX, respectively. Statistical significance was defined as 95% CI not crossing zero. Time points are shown relative to osteochondral fragment induction. Table showing only parameters and time points were differences were observed.

Parameter	Time	Comparison vs NeoSTX	Lower CI	Upper CI	Significant (95% CI)
IFN-γ	Day 20	Saline	-38,5	-3,26	YES
		Triamcinolone	-159,4	-4,42	YES
IL-10	Day 20	Saline	-55,8	14,81	NO
		Triamcinolone	-101	-13,9	YES
	Day 30	Saline	-54,5	16,17	NO
		Triamcinolone	-141,3	-54,2	YES
IL-17	Day 30	Saline	-0,34	-0,24	YES
		Triamcinolone	-0,46	0,34	NO
IL-18	Day 20	Saline	-1,43	0,08	NO
		Triamcinolone	-10,46	-0,92	YES
	Day 30	Saline	-1,76	-0,26	YES
		Triamcinolone	-7,47	2,07	NO
IL-1β	Day 20	Saline	-32,1	-3,7	YES
		Triamcinolone	-130	105,91	NO
	Day 30	Saline	-26	2,33	NO
		Triamcinolone	-237	-1,12	YES
IL-4	Day 20	Saline	-2,62	1,49	NO
		Triamcinolone	-39,6	-1,34	YES
IL-6	Day 20	Saline	-241,5	-51,8	YES
		Triamcinolone	-85	13,5	NO
IP10	Day 30	Saline	-0,38	-0,06	YES
		Triamcinolone	-0,04	0,06	NO
PMNn (#)	Day 20	Saline	-5,51	-1,29	YES
		Triamcinolone	-10,73	-1,35	YES
	Day 30	Saline	-4,34	-0,12	YES
		Triamcinolone	-9,63	-0,25	YES
PMNn (%)	Day 30	Saline	-23,03	-0,07	YES
		Triamcinolone	-25,4	18,3	NO
RBC	Day 10	Saline	-0,47	0,3	NO
		Triamcinolone	-0,56	-0,037	YES
	Day 20	Saline	-0,94	-0,17	YES
		Triamcinolone	-0,35	0,17	NO
Total Proteins	Day 20	Saline	-2,04	-0,45	YES
		Triamcinolone	-1,42	0,4	NO
	Day 30	Saline	-1,7	-0,11	YES
		Triamcinolone	-1,23	0,59	NO

Table S2. Multiple between-group comparisons demonstrating the comparable behavior of both NeoSTX-treated joints throughout the study, together with comparisons between the TA- and saline- treated joints, including corresponding significant p-values.

Parameter	Time	NEO(t) vs NEO(c) p-value	NEO(c) vs Saline p-value	TA vs Saline p-value	NEO(t) vs TA p-value
**PMN**	baseline	0,45			
	day 10	0,99			
	day 20	0,26	0,09		
	day 30	0,14	0,048	0,02	
**TP**	baseline	0,89			
	day 10	0,99			
	day 20	0,84	0,008		
	day 30	0,84	0,013		
**MCP1**	baseline	0,99			
	day 10	0,55		0,03	
	day 20	0,43			
	day 30	0,48	0,05		
**IP10**	baseline	0,95			
	day 10	0,83			
	day 20	0,17	0,014	0,01	
	day 30	0,24	0,009	0,001	
**IL1b**	baseline	1			
	day 10	1			
	day 20	1	0,018		
	day 30	1	0,099	0,06	0,02
**IL2**	baseline	0,99			
	day 10	0,41			
	day 20	0,99			0,06
	day 30	0,99	0,07		0,055
**IL4**	baseline	1			
	day 10	0,8			
	day 20	0,99		0,07	0,03
	day 30	0,99			
**IL6**	baseline	1			
	day 10	0,85			
	day 20	1	0,031	0,04	
	day 30	1	0,017		
**IL10**	baseline	0,42			
	day 10	1			
	day 20	0,98			0,01
	day 30	0,97		0,04	0,001
**IL18**	baseline	0,99			
	day 10	0,99			
	day 20	0,99	0,07	0,05	0,02
	day 30	0,99	0,01		
**IFN**	baseline	0,99			
	day 10	0,99			
	day 20	0,99	0,002		0,038
	day 30	0,98			
**TNF**	baseline	1			
	day 10	0,68			
	day 20	0,94			0,078
	day 30	0,97			
**Neutrophils**	baseline	0,99			
	day 10	1	0,021	0,025	0,013
	day 20	1			
	day 30	0,99	0,038		0,039
**RBC**	baseline	0,97			
	day 10	0,97			
	day 20	0,99	0,006	0,025	
	day 30	0,14	0,014		
